# Unraveling the enigma of B cells in diffuse large B-cell lymphoma: unveiling cancer stem cell-like B cell subpopulation at single-cell resolution

**DOI:** 10.3389/fimmu.2023.1310292

**Published:** 2023-12-11

**Authors:** Fengling Liu, Jie Zheng, Gaohui Yang, Lin Pan, Yanni Xie, Siyu Chen, Jinwei Tuo, Jinxia Su, Xiuyi Ou, Rongrong Liu

**Affiliations:** ^1^ Department of Hematology, The first Affiliated Hospital of Guangxi Medical University, Nanning, China; ^2^ Guangxi Key Laboratory for Genomic and Personalized Medicine, Guangxi Collaborative Innovation Center for Genomic and Personalized Medicine, Guangxi Medical University, Nanning, China

**Keywords:** diffuse large B cell lymphoma, cancer stem cell, single-cell RNA sequencing, bulk RNA sequencing, transcription factor, immune escape

## Abstract

**Background:**

Diffuse large B-cell lymphoma (DLBCL) represents the most prevalent form of aggressive non-Hodgkin lymphoma. Despite receiving standard treatment, a subset of patients undergoes refractory or recurrent cases, wherein the involvement of cancer stem cells (CSCs) could be significant.

**Methods:**

We comprehensively characterized B cell subpopulations using single-cell RNA sequencing data from three DLBCL samples and one normal lymph tissue. The CopyKat R package was employed to assess the malignancy of B cell subpopulations based on chromosomal copy number variations. CIBERSORTx software was utilized to estimate the proportions of B cell subpopulations in 230 DLBCL tissues. Furthermore, we employed the pySCENIC to identify key transcription factors that regulate the functionality of B cell subpopulations. By employing CellphoneDB, we elucidated the interplay among tumor microenvironment components within the B cell subpopulations. Finally, we validated our findings through immunofluorescence experiments.

**Results:**

Our analysis revealed a specific cancer stem cell-like B cell subpopulation exhibiting self-renewal and multilineage differentiation capabilities based on the exploration of B cell subpopulations in DLBCL and normal lymph tissues at the single-cell level. Notably, a high infiltration of cancer stem cell-like B cells correlated with a poor prognosis, potentially due to immune evasion mediated by low expression of major histocompatibility complex molecules. Furthermore, we identified key transcription factor regulatory networks regulated by *HMGB3*, *SAP30*, and *E2F8*, which likely played crucial roles in the functional characterization of the cancer stem cell-like B cell subpopulation. The existence of cancer stem cell-like B cells in DLBCL was validated through immunofluorescent staining. Finally, cell communication between B cells and tumor-infiltrating T cell subgroups provided further insights into the functional characterization of the cancer stem cell-like B cell subpopulation.

**Conclusions:**

Our research provides a systematic description of a specific cancer stem cell-like B cell subpopulation associated with a poor prognosis in DLBCL. This study enhances our understanding of CSCs and identifies potential therapeutic targets for refractory or recurrent DLBCL patients.

## 1 Introduction

Non-Hodgkin’s lymphoma ranks among the top ten most common malignancies affecting both males and females, accounting for 2.8% of all new tumor cases and 2.6% of all tumor deaths worldwide in 2020 (1). The most prevalent subtype, diffuse large B-cell lymphoma (DLBCL), makes up 40% of all non-Hodgkin’s lymphoma cases (2). R-CHOP regimen, the standard chemoimmunotherapy treatment of DLBCL, only can lead to a cure in around 50%-60% of patients, and the rest of the refractory or relapse patients usually have poor outcomes (3). Therefore, there is an urgent need to gain insight into the underlying mechanisms of refractory or relapsed DLBCL to identify promising therapeutic approaches.

Cancer stem cells (CSCs) are a distinct group of cells with self-renewal and multidirectional differentiation capabilities, which are closely associated with drug resistance, recurrence or refractoriness, and poor prognosis in tumor patients (4, 5). The concept of CSCs was first hypothesized in acute myeloid leukemia, and then validated in many solid tumors, including liver cancer, breast cancer, kidney cancer, bladder cancer, colorectal cancer, and so on (6). For instance, Pan et al. (7) utilized high-resolution single-cell technology to deeply analyze the heterogeneity of tumor cells in collecting duct renal cell carcinoma, leading to the discovery of a subgroup of CSCs closely associated with tumor bone metastasis, which provided a new perspective for targeted therapies in this highly malignant tumor. In the context of DLBCL, the previous report demonstrated that the knockdown of *ZNF267* (a gene positively correlated with CSCs) decreased the stemness characteristics of DLBCL and impeded its proliferation and metastasis (5). Chen et al. (8) established a direct link between the presence of CSCs and resistance to R-CHOP by developing drug-resistant DLBCL cell lines. Their findings also highlighted the potential of targeting core stemness-associated transcription factors, such as *SOX2*, which could reduce the survival of CSCs and potentially reverse drug resistance. However, although recent research has preliminarily suggested the existence of CSCs in DLBCL, there is currently a dearth of comprehensive research on CSCs in DLBCL (9, 10), particularly in the realm of omics-based investigations. With the advancements in single-cell genomics, it is now possible to dissect the heterogeneity of cells within tumors at high resolution and uncover rare cellular subpopulations (11, 12). Gaining a deeper understanding of the molecular characteristics of CSCs in DLBCL will be instrumental in unraveling the underlying mechanisms of refractory or relapsed DLBCL, thereby identifying promising therapeutic approaches.

In this study, we aimed to integrate single-cell RNA sequencing (scRNA-seq) data and bulk RNA-seq data to reveal the transcriptome characterize and clinical value of cancer stem cell-like B cells in DLBCL. In addition, we would focus on the key transcription factor regulatory networks that regulated the functional characterization of cancer stem cell-like B cell subgroup. The study would improve our understanding of the characterize of cancer stem cell-like B cells and provide new therapeutic ideas for relapsed and refractory DLBCL patients.

## 2 Materials and methods

### 2.1 Data acquisition

In this study, we utilized scRNA-seq data from a study by Steen et al. (13) for further analysis, which could be downloaded from the Gene Expression Omnibus database (GEO, https://www.ncbi.nlm.nih.gov/geo/, GSE182436), including three cases of DLBLC tumors (DLBCL002, DLBCL007, and DLBCL111) and a patient with tonsilitis (represented the normal lymph tissue). Among these cases, DLBCL002 and DLBCL111 samples were obtained from patients diagnosed with activated B-cell (ABC) molecular subtype of DLBCL, while DLBCL007 sample was obtained from a patient diagnosed with germinal center B-cell (GCB) molecular subtype. After filtering out samples with missing or less than 1 month of follow-up information, we obtained 230 DLBCL tumor samples from the Genomic Data Commons Data Portal (GDC Data Portal, https://portal.gdc.cancer.gov/) for further bulk-RNA sequencing analysis.

### 2.2 scRNA-seq data processing

The Seurat R package (version 4.1.0) (14) was extensively utilized for the scRNA-seq data processing, encompassing crucial tasks such as data normalization, dimensionality reduction, clustering, and visualization. To ensure data quality, rigorous quality control measures were implemented, including the exclusion of cells with total gene expression below 200 or above 5000, as well as cells exhibiting mitochondrial RNA content exceeding 10%. To address batch effects among the four samples, the harmony R package (version 1.0) (15) was applied for data integration. Differential gene expression analysis between subpopulations was performed using the FindAllMarkers function in Seurat, employing a significance threshold of P-adjust < 0.05. The standardized markers were then used for the annotation of cell types. In the reanalysis of B or T cell subpopulations, the same approach as described above was followed, while excluding the need for additional quality control steps.

### 2.3 Identification of tumor malignant cells and single-cell copy number variation analysis

The CopyKat R package (16) was used to analyze copy number variations (CNV) in B cells obtained from three tumor samples, using B cells from the normal lymph tissue as reference cells. Moreover, this package was utilized to distinguish between malignant and benign B cells within the tumor samples. The benign cells are characterized by diploid copy number, while malignant cells typically exhibit aneuploid copy number variations. The basic parameters of CopyKat are set as follows: ngene.chr = 5, win.size = 25, KS.cut = 0.15, distance = “euclidean”.

### 2.4 Pseudotime trajectory analysis

The development trajectories of B and T cells were performed by the Monocle 3 R package (version 0.2.3.0) (17). Moreover, the analysis process was standardized and processed using the functions provided by the Monocle 3 R package. To enhance our understanding of the development trajectories between subpopulations, we utilized the Seruat R package (version 4.1.0) (14) to visualize the pseudo-temporal trajectory of uniform manifold approximation and projection (UMAP) coordinates.

### 2.5 CIBERSORTx analysis

CIBERSORTx, known as “digital flow cytometry”, is a powerful tool developed for quantifying the abundance of cell types in bulk RNA-seq data (18). In our study, we utilized this tool to assess the infiltration levels of various B cell subpopolations in a cohort of 230 DLBCL patients. To achieve this, we deconvolved the bulk RNA-seq data using the CIBERSORTx Docker image and chosen the CIBERSORTx Fractions module. The input data were derived from the expression matrices of scRNA and bulk-RNA.

### 2.6 Survival analysis and Cox analysis

The comprehensive analysis was conducted for incorporating Kaplan-Meier (KM) survival analysis, as well as univariate and multivariate Cox regression analysis, utilizing the survminer and survival R packages. The single-sample Gene Set Enrichment Analysis (ssGSEA) algorithm from the GSVA R packages (version 1.42.0) (19) was used to score characteristic genes of cluster 4 in bulk-RNA data. Because of the sparsity of the single-cell matrix, we considered both the multiple of average expression difference and the proportion of gene expression in the subset as constraints for the selection of cluster 4 characteristic genes (p<0.05, the proportion of gene expression in the cluster 4 > 0.8, the proportion of gene expression in the other cluster<0.5).

### 2.7 SCENIC analysis

To investigate the key transcription factor regulatory networks that regulate the function of different B cell subpopulations, we utilized the Single-Cell reEgulatory Network Inference and Clustering in Python (pySCENIC) for analysis. We computed regulon activity scores (RAS) to determine the activity levels of transcription factors. Then we converted the RAS values into a binary format, generating binarized regulon activity scores (binRAS). The AUCell_exploreThresholds function from the AUCell R package was employed to transform the regulonAUC matrix into a binary matrix. To define active regulatory elements specific to each subpopulation, we set the “smallestPopPercent” parameter to 0.25. This transformation facilitated easier interpretation and analysis of the activity states of transcription factors in different B cell subpopulations. The conversion involved Z score normalization across all cells based on the RAS values and setting a threshold as a cutoff. A value of 1 denoted an active state, while a value of 0 indicated an inactive state. Moreover, we calculated the regulatory specificity score (RSS) for each B cell subpopulation using the calcRSS function from the SCENIC R package (version 1.3.1) (20). The RSS provided a quantitative measure of the regulatory activity specific to each subpopulation.

### 2.8 Cell-cell communication analysis

We performed additional analysis to estimate cell-cell interaction levels between different subpopulations using the Python-based CellPhoneDB (21). The corresponding standardized data matrix was incorporated into the analysis. Ligand-receptor pairs with p>0.05 calculated by CellPhoneDB were filtered.

### 2.9 Functional enrichment analysis

We conducted functional enrichment analysis on differentially expressed genes obtained from different subpopulations using the enricher function from the clusterProfiler R package (version 4.2.2) (22). For the functional enrichment analysis, we downloaded 50 hallmark reference gene sets from the Molecular Signatures Database (MSigDB, https://www.gsea-msigdb.org/gsea/msigdb). The enrichKEGG and enrichGO functions were used to perform KEGG and GO feature enrichment analysis, respectively.

### 2.10 Immunofluorescent staining

Paraffin-embedded DLBCL sections confirmed by pathologists were collected. Paraffin-embedded DLBCL sections were deparaffinized with the dewaxing agent and absolute ethanol. After that, citric acid solution (Servicebio, G1202) was used for antigen retrieval and 3% H2O2 was used to inactivate endogenous peroxidase. Then 3% bovine serum albumin (Servicebio, GC305010) was added to block sections for 30 minutes. Add the primary antibody, anti-*CD79A* (ZEN BIO, R23860), and incubate overnight at 4 °C. After washing sections three times using PBS, add goat anti-rabbit IgG H&L (HRP) (Servicebio, GB21303) and incubate for 50 minutes. After washing three times using PBS, add CY3-tyramide (Servicebio, G1223) for 10 minutes. Wash with a citric acid solution to remove the first type of primary antibody. Then add the second primary antibody, anti-*HMGB3* (Affinity Biosciences, AB_2841269), and incubate overnight at 4 °C. After washing sections three times using PBS, add anti-rabbit IgG (Alexa Fluor 488 conjugate) (Servicebio, GB25303) and incubate for 50 minutes. Finally, sections were stained with DAPI (Servicebio, G1012) and sealed with an anti-fluorescent quenching agent (Servicebio, G1401). Finally, fluorescence image capture was performed using laser scanning confocal microscopy and processed using ImageJ software.

### 2.11 Statistical analysis

All correlation was assessed using the Spearman Rank Correlation test. The comparison of exhaustion scores among T cell subsets was evaluated by the Kruskal-Wallis test. A value of p < 0.05 was considered statistically significant in all statistical analysis.

## 3 Results

### 3.1 Single-cell transcriptomic atlas of DLBCL and normal lymph tissues

**Figure 1:**
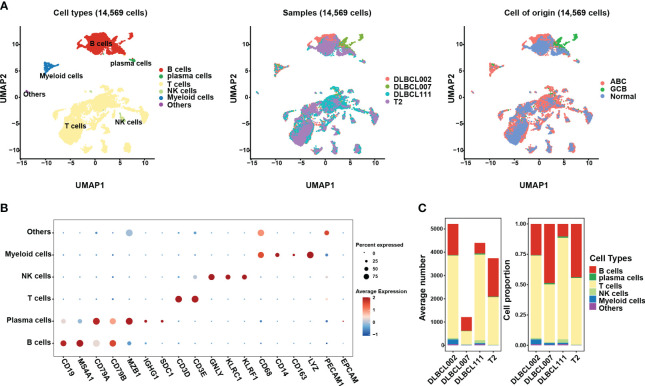
The single-cell transcriptomic atlas of one normal lymph tissue and three DLBCL samples. **(A)** The left panel shows a UMAP visualization of six major cell clusters in the scRNA-seq data from four samples. The middle and right panels provide an overview of these six major cell clusters, categorized by sample cases and DLBCL molecular subtypes. Specifically, DLBCL002 and DLBCL111 belong to the ABC subtype, DLBCL007 belongs to the GCB subtype, and T2 represents normal lymph tissue. **(B)** The dot plot depicts the expression patterns of marker genes for each cell type in the scRNA-seq data. The size of the dots represents the percentage of gene expression within the cell subgroups, while the color indicates the intensity of expression. **(C)** The average number (left panel) and cell proportion (right panel) of six major cell clusters in four samples.

In this study, we investigated scRNA data from four tissue samples, including three samples from patients with DLBCL (two of the ABC subtype and one of the GCB subtype) and one sample from normal lymph tissue (**Figures 1A**, **S1A**). In order to ensure data quality, rigorous quality control measures were applied, resulting in a total of 14,569 cells deemed suitable for further analysis (**Figures S1B-S1C**). To address potential batch effects between the different samples, the scRNA data from the four different samples were integrated using the Harmony R package (15), which aided in minimizing any unwanted variations introduced by technical differences (**Figures 1A**, **S1A**).

By leveraging classic marker genes (23), we successfully identified distinct cell types within the integrated scRNA data. This strategy facilitated the classification of cells into six main subpopulations, each exhibiting their own set of characteristic marker genes (**Figures 1A, B**). These subpopulations included a B cell cluster (marked by *CD19*, *MAS4A1*, *CD79A*, and *CD79B*), a plasma cell cluster (marked by *MZB1* and *IGHG1*), a T cell cluster (marked by *CD3D*, *CD3E*), an NK cell cluster (marked by *GNLY*, *KLRC1*, and *KLRF1*), and a myeloid cell cluster (marked by *CD68*, *CD14*, *CD163*, and *LYZ*). However, there exists a population of cells that exhibit concurrent expression of markers associated with both immune cells and endothelial cells, yet there is presently no definitive description or nomenclature for this cell subset. These cells were grouped together in the “others cluster” (**Figure 1B**). Among the identified cell subpopulations, T cells were found to be the main cluster across all samples, followed by the B cell cluster (**Figure 1C**). It is worth noting that the proportion of B cells within tumor samples varied, indicating the presence of heterogeneity within the tumor samples (**Figure 1C**).

### 3.2 Refined analysis of B cell subgroups revealed tumor cell heterogeneity and identified a cancer stem cells like B cell subgroup

**Figure 2:**
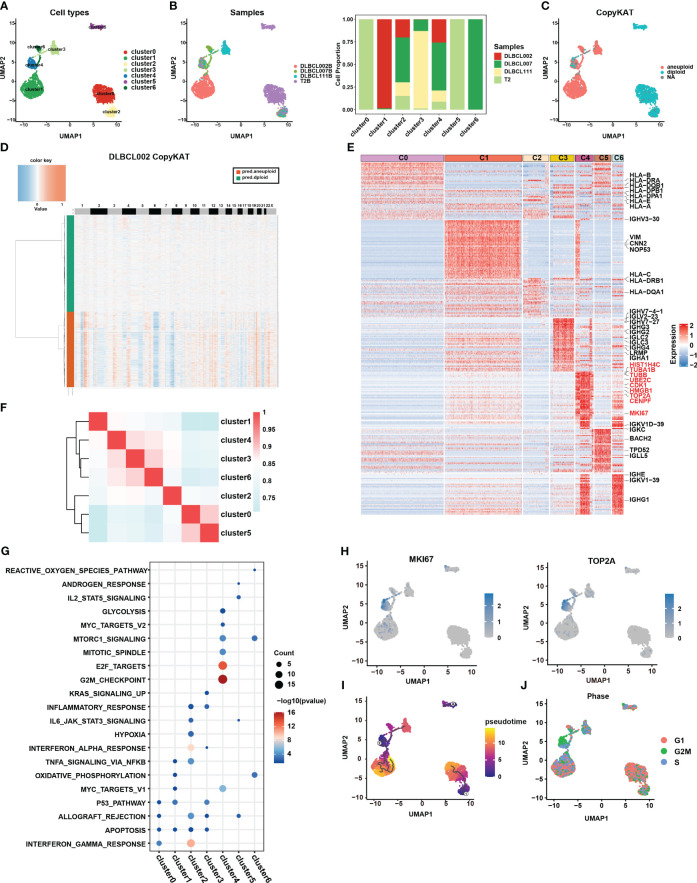
Re-clustering of B cells in one normal lymph tissue and three DLBCL samples. **(A)** UMAP visualization of seven B cell subsets in four samples after re-clustering B cells. Renaming cell subpopulations to cluster0, cluster1, cluster2, cluster3, cluster4, cluster5, and cluster6. Different cell populations are marked with different colors, with one dot representing one cell. **(B)** The left panel displays the overview of six major cell clusters categorized by sample cases. The right panel presents a stacked histogram illustrating the cell proportion of sample types in seven B cell subsets. **(C)** The UMAP visualization depicts the distinction between malignant and benign types within the seven B cell subsets, achieved through the utilization of copyKAT. In this context, the term “aneuploid” represents malignant cells, “diploid” represents benign cells, and “NA” indicates undefined cases. **(D)** The chromatin deletion and amplification in benign (green) and malignant (orange) B cell subsets in the DLBCL002 sample. In the figure, the rows represent cells, and the columns represent the positions of chromosomes. The presence of chromosomal deletions and amplifications in the cells is depicted using a gradient of blue and red colors. The more inclined towards blue, the greater the extent of chromosomal deletions, while the more inclined towards red, the greater the extent of chromosomal amplifications. **(E)** Heatmap of specific differential expression genes in seven B cell subsets. **(F)** The correlation analysis of different B cell subtypes is presented. The color represents the strength of the correlation. **(G)** Gene enrichment analysis was performed on the differentially expressed genes of the seven B cell subsets using the Hallmark gene sets. **(H)** UMAP showing the expression of *MKi67* and *TOP2A* in seven B cell subsets. **(I)** The trajectory analysis of the seven B cell subsets was conducted using Monocle3. A lower value of pseudotime indicates a higher proliferative and differentiation potential. **(J)** Cell cycle analysis was performed on the seven B cell subsets. In this analysis, the G1 phase is represented by red, the G2/M phase by green, and the S phase by blue.

To gain a deeper understanding of the tumor heterogeneity in DLBCL, we conducted a comprehensive analysis of the B-cell subsets. Our analysis identified a total of 4,032 B cells across the four samples. Despite using the harmony R package (15) to eliminate batch effects, the sample distribution of cell subsets still exhibited marked heterogeneity (**Figures 2A, B**). For better comprehension, we defined the seven clusters obtained after refined analysis of B cell subgroups as clusters 0-6 (**Figure 2A**). Among them, clusters 0 and 5 were derived entirely from the normal tissue sample, T2B, while clusters 1, 3, and 6 primarily originated from distinct tumor tissue samples, namely DLBCL002B, DLBCL111, and DLBCL007, respectively (**Figure 2B**). The results demonstrated the significant tumor tissue heterogeneity. It was worth noting that, despite the significant heterogeneity presented, cluster 2 and cluster 4 still contained four samples of B cells (**Figures 2A, B**).

CopyKAT is a method used to distinguish between benign and malignant cells based on their CNV (16). Benign cells are characterized by diploid copy number, while malignant cells typically exhibit aneuploid copy number variations. Here, we utilized this method to discriminate between benign and malignant B cells in the four samples. The results indicated that all B cells from the normal tissue sample (primarily clusters 0 and 5), as well as a small fraction of B cells from three tumor tissue samples (cluster 2), were categorized as benign cells (**Figure 2C**). In contrast, the majority of B cells originating from tumor samples were classified as malignant cells, specifically clusters 1, 3, and 6 (**Figure 2C**). These cells exhibited clear chromatin deletion and amplification, indicating significant genomic alterations associated with malignancy (**Figures 2D**, **S2A**). It is worth noting that cluster 4 encompassed both benign and malignant cell types, warranting further investigation to understand its characteristics and behavior (**Figure 2C**).

To gain a deeper understanding of the functionality and biological characteristics of different B cell subgroups, we compared the transcriptomic features among these subgroups (**Figure 2E**) (**Table S1**). Among them, cluster 0 and cluster 2, defined as benign B cells, expressed high levels of MHC molecules gene expression (such as *HLA-B*, *HLA-DPA1*, *HLA-DPB1*, *HLA-E*, *HLA-A*, and *HLA-DRA*) and enrichment in pathways related to antigen presentation and interferon (**Figures 2E, G**, **S2B**). In addition, based on correlation analysis, we observed that the transcriptional profiles of cluster 0 and cluster 5 derived from normal tissues exhibited a higher degree of similarity with the transcriptional profile of cluster 2, which contains cells from tumor samples, compared to other cell subsets primarily derived from tumor samples (**Figure 2F**). Furthermore, clusters 3 displayed higher levels of IgG molecule expression, which may indicate a more mature state (**Figure 2E**). However, the most surprising finding was the discovery of cluster 4, which exhibited specific expression of numerous genes related to cell proliferation, cycle, and stemness, such as *MKI67*, *TOP2A*, *TUBA1B*, *TUBB*, *UBE2C*, *HMGB1*, *CENPF*, and *CDK1* (**Figures 2E, H**). This expression pattern closely resembled the characteristic gene expression of CSCs subset identified in collecting duct renal cell carcinoma by Pan et al. (7).

Furthermore, the results of functional enrichment analysis also revealed that cluster 4 exhibited significant enrichment in cell cycle and stem cell-related pathways, including G2M checkpoint, E2F targets, MYC targets V1, MYC targets V2, mTORC1 signature, and mitotic spindle-related pathways (**Figures 2G**, **S2B**). Notably, pseudotime analysis indicated that cluster 4 had a lower pseudotime value (**Figure 2I**), demonstrating its multilineage differentiation capabilities. Additionally, cell cycle analysis further demonstrated that most cells in cluster 4 were arrested at the G2M phase (**Figure 2J**), implying active cell proliferation within this cluster. Combining the above results, we considered that the cluster 4 subpopulation may be closely related to stemness differentiation. It was worth noting that cluster 4 also contained B cells from the normal sample, which to some extent reflects that there were a small number of cells with possible malignant transformation in the normal sample (**Figure 2B**). This further highlighted the significance of this specific subpopulation of cells in DLBCL.

### 3.3 Low MHC molecule expression in cancer stem cell-like B cell subgroup correlated with a poor prognosis in DLBCL

**Figure 3:**
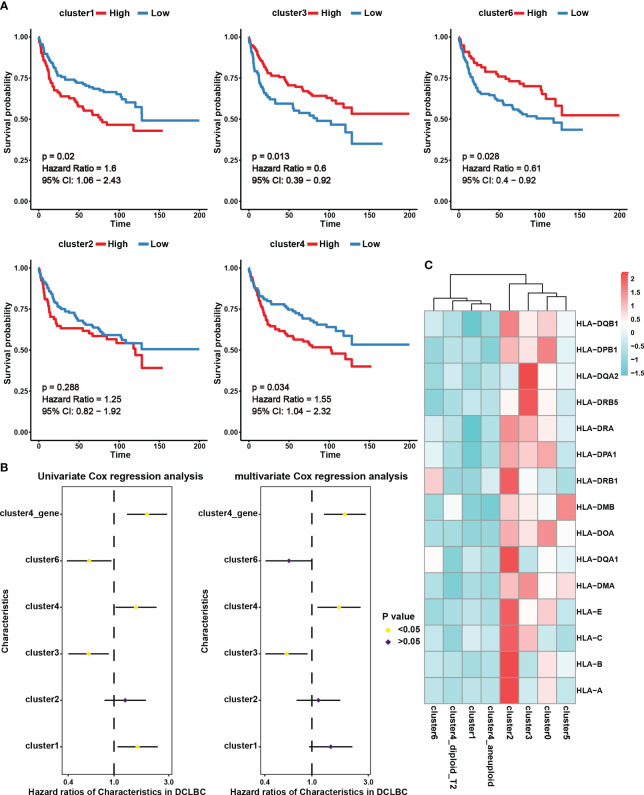
The prognostic effect and expression characteristics of MHC molecules in the seven B cell subsets. **(A)** Survival analysis for DLBCL derived B cell subset infiltration levels in 230 tumor samples from the Genomic Data Commons Data Portal. The infiltration levels of the B cell subsets in the 230 samples were categorized into high infiltration group (represented by red color) and low infiltration group (represented by blue color). **(B)** Univariate and multivariate Cox analysis for DLBCL derived B cell subset infiltration levels. Among them, cluster1, cluster2, cluster3, cluster4, and cluster6 represent the infiltration levels of B cell subsets in 230 samples. These levels were obtained through deconvolution using the CIBERSORTx algorithm based on single-cell data. On the other hand, cluster4_gene represents the scores calculated using the ssGSEA algorithm for specific genes associated with the cluster4 subset across the 230 samples. **(C)** Average expression of major histocompatibility complex (MHC) molecules in seven B cell subsets. The color indicates the intensity of the expression.

In this study, we assessed the abundance of different B cell subgroups in 230 DLBCL samples from the GDC database using CIBERSORTx (18). Cluster 0 and cluster 5 were excluded from further analysis as they predominantly originated from the normal sample (**Figure 2B**). KM survival analysis revealed that the abundance of cluster 1 and cluster 4 was associated with a poor prognosis, whereas cluster 3 and cluster 6 were indicative of a good prognosis (**Figure 3A**, p < 0.05). Univariate Cox analysis further validated the results of KM survival analysis (**Figure 3B**). However, after eliminating interference factors such as gender, age, and staging, multivariate Cox analysis suggested that only the infiltration abundance of cluster 3 and cluster 4 had an impact on patients’ survival (**Figure 3B**, p < 0.05). Moreover, it is noteworthy that we performed ssGSEA scoring on the characteristic gene sets of clusters 4 in DLBCL patients (**Table S4**), and both univariate and multivariate Cox analysis suggested its correlation with a poor prognosis in patients (**Figure 3B**, p < 0.05). This further suggested that cluster 4 subgroup was associated with a poor prognosis in DLBCL patients.

We further analyzed MHC molecule expression levels in different cell subsets to explore differences in antigen presentation and immune activity. According to our results, cluster 4 and cluster 1, which were linked to a poor prognosis, exhibited lower expression of MHC molecules, potentially contributing to immune evasion (**Figure 3C**). Interestingly, in addition to the analysis results showing high expression of MHC molecules in benign cell clusters 0 and 2, we also observed high levels of MHC expression in the malignant cell cluster, cluster 3 (**Figure 3C**). This indicates that these malignant cells have an advantage in preserving effective antigen presentation function, which may be an important favorable factor for prognosis.

### 3.4 Identifying key transcription factor regulatory networks of cancer stem cell-like B cell subgroup

**Figure 4:**
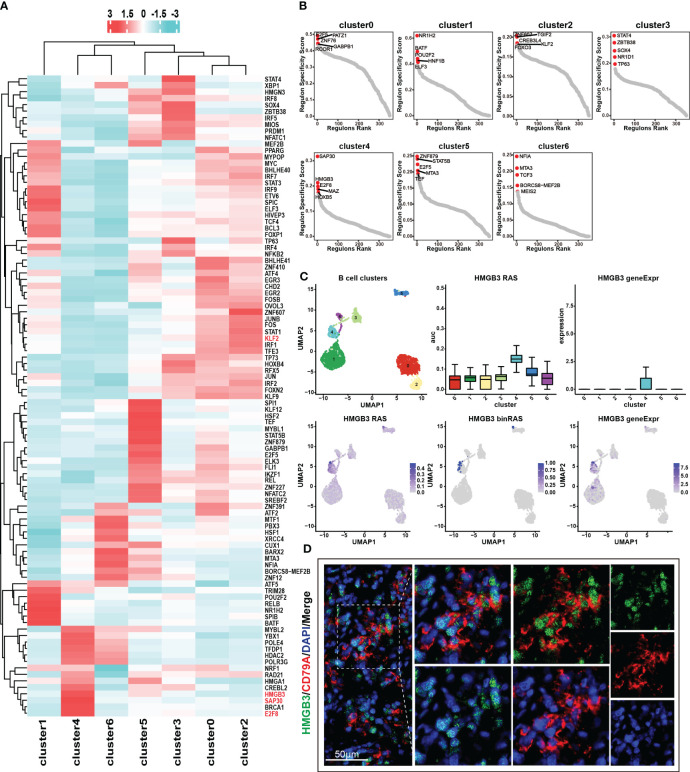
Transcription factor regulatory networks in seven B cell subsets. **(A)** Top 50 transcription factor regulatory networks in seven B cell subsets. Color indicates the value of RAS (regulon activity scores). **(B)** RSS (regulon specificity score) of transcription factor regulatory networks in seven B cell subsets and the top 5 transcription factors were marked in the graph. A higher RSS value indicates greater specificity of the transcription factor within that particular subset. **(C)** Expression and RAS of *HMGB3* in seven B cell subsets. Here, RAS represents Regulon Activity Scores, calculated by pySCENIC. On the other hand, binRAS stands for Binarized Regulon Activity Scores, where Z-score normalization is performed over all cells based on RAS, and a threshold is set as a cutoff to convert the scores to 0 and 1. The threshold is calculated using the AUCell_exploreThresholds function in the SCENIC R package. In comparison to RAS, binRAS allows for the differences in the activity of transcription factors between different cells to be more pronounced. In addition, geneExpr refers to the transcriptional expression of genes. **(D)** Immunofluorescence staining of the characteristic transcription factor, *HMGB3* (green), and the marker gene, *CD79A* (red).

Transcription factors control gene expression by binding to specific DNA sequences, either promoting or inhibiting the transcription of target genes, thereby determining the functions and characteristics of cells. Through the utilization of the pySCENIC analysis (24), we obtained the particular transcription factor regulatory networks operating within distinct cell subgroups. In cluster 4, we found that the transcription factor regulatory networks controlled by transcription factors *SAP30*, *HGMB3*, and *E2F8* exhibit higher levels of regulon activity scores (RAS) (**Figure 4A**) (**Table S2**). Furthermore, through the calculation of the regulatory specificity score (RSS), it has been determined that these three transcription factors have the strongest degree of regulatory specificity within cluster 4 (**Figure 4B**) (**Table S3**). This observation underscores their pivotal role in governing cellular functions and characteristics within cluster 4. Additionally, the results revealed a strong positive correlation between the expression levels of *SAP30*, *HGMB3*, and *E2F8*, and the infiltration abundance and characteristic gene set scores of cluster 4 (**Figures S3A-3C**). However, these correlations showed a negative or non-existent relationship with other subpopulations (**Figures S3A-3C**). The results suggested that the transcription factors *SAP30*, *HGMB3*, and *E2F8* could potentially serve as dependable indicators of the infiltration abundance and transcriptional characteristics of cluster 4 to a certain extent. We conducted further exploration of the expression patterns and activity of these three transcription factors across different cell subpopulations and observed specific expression of *HMGB3* in cluster4 (**Figures 4C**, **S3D-E**). Consequently, we selected *HMGB3* as the specific marker for identifying the cancer stem like-B cell subpopulation and validated it by immunofluorescence staining (**Figure 4D**).

In our previous discussion, we identified similarities in terms of transcriptional and function enrichment features between cluster 0 and cluster 2 (**Figures 2F, G**, **S2B**). Furthermore, we observed that these two benign cell subpopulations also exhibited comparable activities in their transcription factor regulatory networks (**Figure 4A**). We speculate that cluster 2, as a benign B cell subset containing cells from tumor samples, may possess unique mechanisms to prevent malignant transformation and malignant proliferation even under the influence of the tumor microenvironment. Remarkably, our analysis revealed an intriguing finding regarding the transcription factor regulatory network mediated by *KLF2*, which may exhibit a specific role in governing the functional characteristics of cluster 2. We observed the highest RAS of *KLF2* in cluster 2, followed by the benign subgroup, cluster 0 (**Figure S3F**). In contrast, cluster 4 and cluster 6, which represent malignant cell subgroups, showed the lowest RAS levels of *KLF2* (**Figure S3F**). The downregulation of *KLF2* in these malignant cell subgroups may contribute to their increased proliferative capacity. Previous studies have reported that *KLF2* downregulation is a prerequisite for mature B cell activation and proliferation (25). Together, these findings further supported the notion that *KLF2* expression may be a critical factor influencing the maintenance of the benign characteristics of cluster 2 and the high proliferative activity of cluster 4.

### 3.5 Crosstalk between B cell and tumor-infiltrating T cells in DLBCL

**Figure 5:**
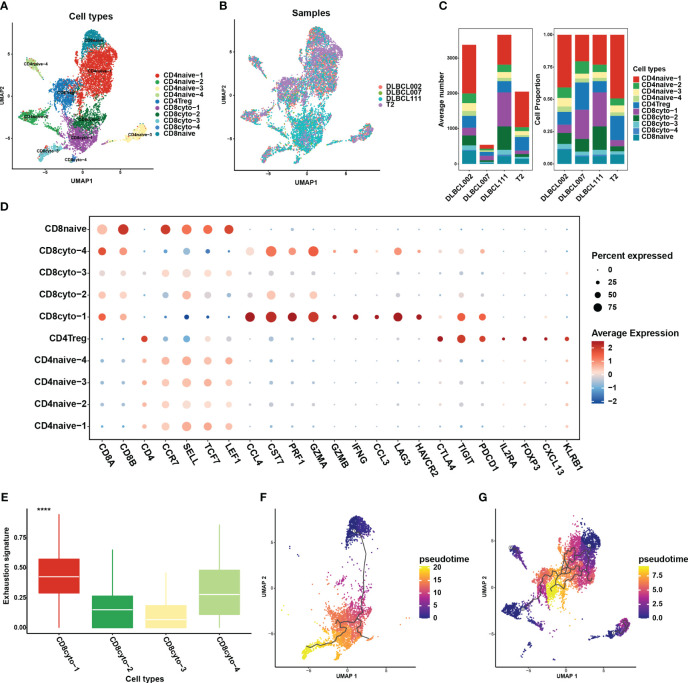
Re-clustering of T cells in one normal lymph tissue and three DLBCL samples. **(A)** UMAP visualization of ten T cell subsets in four samples after re-clustering T cells. Different cell populations are marked with different colors, with one dot representing one cell. **(B)** The panel displays the overview of ten major cell clusters categorized by sample cases. **(C)** The average number (left panel) and cell proportion (right panel) of ten major cell clusters in four samples. **(D)** The dot plot depicts the expression patterns of marker genes for each T cell type in the scRNA-seq data. The size of the dots represents the percentage of gene expression within the cell subgroups, while the color indicates the intensity of expression. **(E)** The comparison of exhaustion signature in four cytotoxic CD8+ T cell subsets. **(F)** The trajectory analysis for CD8+ T cell subsets based on Monocle3. **(G)** The trajectory analysis for CD4+ T cell subsets based on Monocle3. A lower value of pseudotime indicates a higher proliferative and differentiation potential.

The absence of MHC molecule expression in cluster 4 indicated that they may evade T cells killing through immune escape. This may be further demonstrated through an analysis of the cellular communication between B cell and tumor-infiltrating T cell subgroups. In this study, T cells constituted the major cell type in each sample, with the highest proportion of cells. We employed unsupervised clustering to re-aggregate T cells, resulting in ten T cell subgroups (**Figure 5A**). Compared to B cells, different subgroups of T cells displayed lower heterogeneity across tumor samples (**Figures 5B, C**). Within CD8+ T cell subgroups, we further classified cells into four cytotoxic CD8+ T cell subgroups (*CCL4*, *CST7*, *PRF1*, *GZMA*, *GZMB*, *IFNG*, *CCL3*) and one naive T cell subgroup (*CCR7*, *SELL*, *TCF7*, *LEF1*) (**Figure 5D**). Among these subgroups, CD8cyto-1 and CD8cyto-4 cell subgroups showed higher exhaustion signs than CD8cyto-2 and CD8cyto-3, indicating more severe exhaustion states (**Figure 5E**). Additionally, we divided CD4+ T cells into CD4+ naive T cells (*CCR7*, *SELL*, *TCF7*, *LEF1*) and CD4+ Treg cells (*IL2A*, *FOXP3*) (**Figure 5D**). We performed pseudotime analysis separately for CD4+ and CD8+ T cells, revealing that both cell types originated from naive cells and underwent differentiation into other cell subgroups (**Figures 5F, G**).

**Figure 6:**
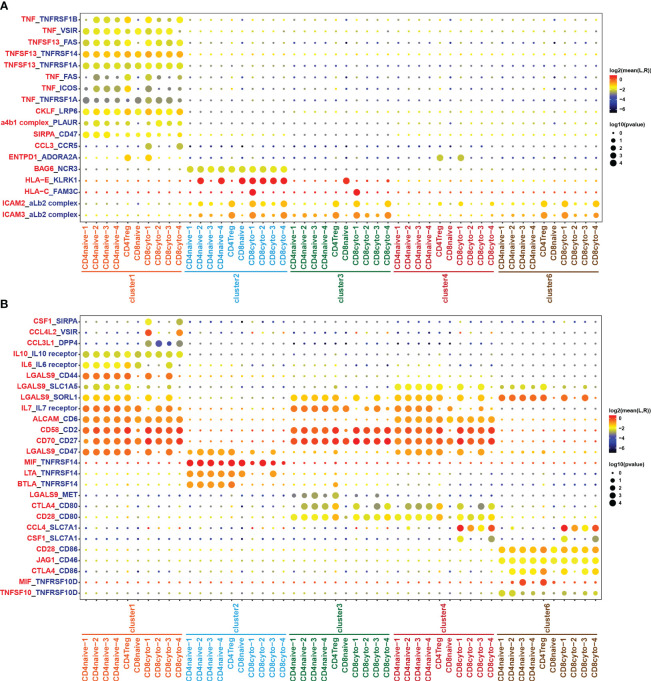
Cell crosstalk between T and B cell subsets. Bubble plot showing the significant ligand-receptor pairs between T and B cell subsets. **(A)** B cell subsets act as ligands, while T cell subsets act as receptors. **(B)** T cell subsets act as ligands, while B cell subsets act as receptors. The color of the circles in the legend represents the average expression intensity of the ligand-receptor pairs, while the size of the circles represents the magnitude of the p-values.

Through comprehensive analysis of cell communication between B cells and tumor-infiltrating T cells, we have identified a significant number of receptor-ligand pairs that were specific to subpopulations (**Figures 6A, B**). Notably, we observed a high abundance of TNF/TNFR ligand-receptor pairs between B cells and tumor-infiltrating T cell subgroups (**Figures 6A, B**). When analyzing B cell subgroups as ligands, we found specific expression of TNF and TNFSF13 ligands in cluster 1, resulting in extensive cell interactions with T cell subgroups (**Figure 6A**). Conversely, in the analysis of B cell subgroups as receptors, we observed the specific expression of the TNFRSF14 receptor in the benign cluster 2, while the TNFRSF10D receptor exhibited characteristic expression in the malignant cluster 6, engaging in cell interactions with T cell subgroups (**Figure 6B**). These results reveal that the TNF/TNFR member family may exert specific effects on the formation of the tumor immune microenvironment in DLBCL through distinct ligand-receptor combinations, thereby impacting tumor development and the prognosis of DLBCL patients. On the other hand, our analysis revealed the presence of HLA-E/KLRK1 and HLA-C/FAM3C ligand-receptor pairs in cluster 2 and cluster 3, while cluster 1 and cluster 4 exhibited no presence (**Figure 6A**), which closely correlated with the loss of MHC molecules in cluster 1 and cluster 4 (**Figure 3C**).

## 4 Discussion

CSCs have been implicated in tumor aggressiveness and drug resistance in various cancers, including DLBCL (5, 6, 8). In this study, we revealed specific cancer stem cell-like B cell subgroup exhibiting self-renewal, multilineage differentiation, and high proliferation capabilities in DLBCL by integrating scRNA-seq and bulk RNA-seq analysis. Meanwhile, we found that the high-level infiltration of cancer stem cell-like B cells was associated with a poor prognosis, potentially due to immune evasion caused by low expression of MHC molecules. Additionally, we identified the key transcription factor regulatory networks regulated by *HMGB3*, *SAP30*, and *E2F8* that may play important roles in the functional characterization of the cancer stem cell-like B cell subgroup. Through cellular communication analysis, we uncovered the interactions between malignant B cells and tumor-infiltrating T cells. The study would provide an in-depth understanding of CSCs and identify promising therapeutic targets for refractory or recurrent DLBLC patients.

DLBCL is a disease characterized by significant heterogeneity, with B cells exhibiting diverse features and variations. Through in-depth research and analysis on B cell subpopulations in DLBCL and normal lymph tissues at the single-cell level, our study revealed a specific cancer stem cell-like B cell subpopulation exhibiting self-renewal and multilineage differentiation capabilities, referred to as cluster 4. It displaying remarkable stem cell-like properties and unique gene expression patterns, which is similarly to the CSCs subpopulation in collecting duct renal cell carcinoma described by Pan et al. (7). Cluster 4 mainly showed enrichment in cell proliferation-related signaling pathways, including the E2F targets, MYC targets v1 and v2, and G2M checkpoint. Notably, these pathways were also significantly upregulated in groups with a high stemness-related prognostic index for head and neck squamous cell carcinomas (26). Among them, the MYC targets v1 pathway was also significantly upregulated in another malignant cell subpopulation, cluster 1, which was associated with poor prognosis along with cluster 4. MYC is one of the most common proto-oncogenes with a wide range of roles in regulating cancer cellular function, including proliferation, differentiation, metabolism, apoptosis, autophagy, aggressiveness, angiogenesis, and immune escape (27). More than half of tumors developed dysfunction of MYC family gene/protein expression (28). As for DLBCL, the prevalence of *MYC* gene amplification was almost 10.41% (27), and numerous studies showed that *MYC* translocations or MYC protein overexpression was a strongly adverse prognostic factor (29–31). Our study further emphasizes the significant impact of the MYC pathway in DLBCL. Additionally, our results demonstrated that the benign cell subgroups, cluster0 and cluster2, were significantly enriched in antigen presentation and interferon-related pathways, including interferon alpha and gamma response. The cluster3 subgroup, associated with a better prognosis, also exhibited significant enrichment in the interferon alpha response pathway. Furthermore, in a study of B-cell acute lymphoblastic leukemia (B-ALL) primarily driven by B-cell malignant transformation, Kumar et al. (32) also reported a deficiency in the secretion of type I interferon by B cells, leading to immune suppression and promoting leukemia development in MYC-driven B-ALL mice. Integrating previous reports with our findings suggests that the interferon-related pathway may also play a role in the immune homeostasis in DLBCL, providing new insights for future treatments.

In addition to similar functional enrichment pathways and their impact on poor prognosis, we also observed low expression of MHC molecular in both cluster 1 and cluster 4. The results of our study infer the view that the poor prognosis may be associated with immune escape. MHC molecules play a central role in the adaptive immune system by presenting foreign peptides for recognition by T cells (33). Low expression levels of MHC-II have also been proven to be associate with poor prognosis through the same mechanism in medullary thyroid cancer and serve as a prognostic biomarker for tumor aggressiveness (34). There is a growing body of evidence suggesting that many cancers can evade immune surveillance by suppressing the expression of MHC-I complex on the cell surface, including osteosarcoma (35), breast cancer (36), pancreatic cancer (37), colorectal cancer (38), endometrial cancer (39), papillary thyroid cancer (40), and lung cancer (41). Fangazio M et al. (42) pointed out that 50% of DLBCL cases exploit immune evasion by degrading MHC-I, thus avoiding the presentation of new tumor antigens to the immune system. Patel N et al. (43) also showed intravascular large B-cell lymphomas achieve immune escape through the downregulation of MHC molecules. Further exploration of the mechanisms leading to the downregulation of MHC molecules may provide insights into ways to reverse this situation and enhance the efficacy of immunotherapy approaches.

Transcription factors play a crucial regulatory role in gene expression by recognizing specific DNA sequences and controlling transcription initiation. Transcription factors and mutations in their binding sites are the major factors contributing to human disease (44). In our study, we identified highly specific and active transcription factor regulatory networks in cancer stem cell-like B cells subset that were predominantly regulated by the three highly expressed transcription factors: *SAP30*, *HGMB3*, and *E2F8.* Our results revealed that these transcription factors could potentially serve as dependable indicators of the infiltration abundance and transcriptional characteristics of cluster 4 to a certain extent. Among them, *HMGB3*, a member of the high mobility group protein family, has been shown to promote the proliferation and invasion of CSCs in laryngeal squamous cell carcinoma by recruiting E2F1 (45). *HMGB3* has also been identified as a specific regulator of the CSC cluster in collecting duct renal cell carcinoma (7). Additionally, elevated levels of *HMGB3* have been linked to the activation of the Wnt/β-Catenin pathway, promoting tumor progression (46, 47). *SAP30* is an integral component of the histone deacetylation complex, and its high expression was associated with a poor prognosis in hepatocellular carcinoma patients (48, 49). Hu et al. (50) demonstrated that *SAP30* can interact with *UHRF1* to promote the activation of the MYC signaling pathway and abnormal self-renewal of leukemia-initiating cells, resulting in the occurrence and development of acute myeloid leukemia and poor prognosis. The *E2F* family affects cell cycle progression, apoptosis, differentiation, and DNA damage repair (51–53). High expression of *E2F8* was related to tumor proliferation and a poor prognosis in a variety of tumors (54–56). Remarkably, our analysis also revealed an intriguing finding regarding the transcription factor regulatory network mediated by *KLF2*, which may be a critical factor influencing the maintenance of the benign characteristics of cluster 2 and the high proliferative activity of cluster 4. *KLF2*, a member of the *KLF* family of transcription factors, plays a crucial role in regulating cellular growth and differentiation. The downregulation of *KLF2* expression is closely associated with the heightened proliferation and invasive capabilities observed in various types of cancer (57). Our findings underscore the importance of transcription factors as key regulators in cancer stem cell-like B cell subpopulation, which may provide new targets for the treatment of DLBCL.

Through the analysis of cell communication, we described the interactions between different B cells and tumor-infiltrating T cells, highlighting the potential role of receptor-ligand pairs formed by members of the TNF/TNFR family. The TNF/TNFR superfamily members, known for their crucial role in immune system function, have been extensively studied in the context of autoimmune diseases, inflammatory diseases, and tumors (58). The combinations of receptor-ligand pairs among different TNF/TNFR family members may have diverse effects on the tumor microenvironment, and these interactions may play a critical role in mediating immune evasion, immune activation, and immune surveillance. In our analysis, we discovered the specific expression of TNF and TNFSF13 ligands in the malignant cluster 1, leading to cellular interactions with T cell subgroups. TNFSF13 was initially reported to be upregulated in cancer cells, including hematologic and solid malignancies (59). Aberrant expression of TNFSF13 may contribute to tumor development through mechanisms such as promoting cell proliferation, inhibiting apoptosis, and influencing signaling pathways in tumor cells (60). The poor prognosis of malignant cluster 1 may be associated with its immune escape mediated by the aforementioned receptor-ligand interactions with T cell subgroups. On the other hand, we found the specific expression of the TNFRSF14 receptor in the benign cluster 2, interacting specifically with T cell subgroups. Previous studies have shown that TNFRSF14 plays a crucial role in the normal functions of humoral and cellular immunity by interacting with ligands such as APRIL and BAFF, thereby promoting the proliferation and activation of T and B cells (61). Cluster 2, as a benign cell subgroup derived from both tumor and normal tissues, may maintain immune homeostasis and immune surveillance, and prevent malignant transformation by interacting with T cells through the corresponding receptor-ligand pairs. Although substantial evidence is still needed to validate our speculation, these findings reveal the critical role of receptor-ligand pairs formed by members of the TNF/TNFR family in DLBCL. Furthermore, our previous discussion emphasized the association between reduced MHC molecule expression and poor prognosis in patients. Our cell communication study further supports this observation by revealing insufficient MHC-mediated communication in the highly malignant clusters 1 and 4. Furthermore, our previous discussions highlighted the association between decreased expression of MHC molecules and unfavorable patient outcomes. This observation is further supported by our cellular communication studies, which revealed insufficient MHC-mediated communication within the highly malignant cluster 1 and cluster 4.

In our study, we performed comprehensive researches on understanding of the molecular characteristics of CSCs in DLBCL, which may provide a considerable reference for the new therapeutic targets for relapsed and refractory DLBCL. However, our article still had certain limitations, and more research was acquired for further confirmation. Firstly, the sample size used for scRNA analysis should be expanded to enhance the persuasiveness of our conclusions. Meantime, refractory or relapsed DLBCL samples should be included for further analysis. Secondly, as a cancer of B cell origin, analysis of the corresponding BCR library will allow us to determine clonal relationships between different cancerous B cells at different stages of differentiation. Lastly, further validation of our conclusions at animal and cellular levels will make our conclusions more reliable. These limitations emphasize the need for future research to address these issues, such as performing BCR repertoire analysis, and validating the molecular characteristics and function of cancer stem cell-like B cells by *in vivo* and *in vitro* experiments, to enhance the robustness and generalizability of our findings.

## 5 Conclusion

In conclusion, cancer stem cell-like B cell subgroup in DLBCL is associated with poor prognosis, potentially due to immune escape, and specific transcription factors in CSCs may become new therapeutic targets for DLBCL patents.

## Data availability statement

The DLBCL scRNA-seq data can be found in the Gene Expression Omnibus database with accession number GSE182436 (https://www.ncbi.nlm.nih.gov/geo/query/acc.cgi?acc=GSE182436). The bulk RNA-seq data were obtained from the Genomic Data Commons Data Portal (https://portal.gdc.cancer.gov/).

## Ethics statement

The studies involving humans were approved by the Medical Ethics Committee of First Affiliated Hospital of Guangxi Medical University. The studies were conducted in accordance with the local legislation and institutional requirements. The human samples used in this study were acquired from the first affiliated hospital of Guangxi Medical University. Written informed consent for participation was not required from the participants or the participants’ legal guardians/next of kin in accordance with the national legislation and institutional requirements.

## Author contributions

FL: Conceptualization, Data curation, Formal analysis, Methodology, Software, Writing – original draft, Writing – review & editing. JZ: Conceptualization, Data curation, Formal analysis, Methodology, Software, Writing – original draft, Writing – review & editing. GY: Resources, Writing – review & editing. LP: Validation, Writing – original draft. YX: Validation, Writing – original draft. SC: Visualization, Writing – original draft. TJ: Visualization, Writing – original draft. JS: Writing – original draft. XO: Writing – original draft. RL: Funding acquisition, Project administration, Supervision, Writing – review & editing.

## References

[1] Sung H Ferlay J Siegel RL Laversanne M Soerjomataram I Jemal A. Global cancer statistics 2020: globocan estimates of incidence and mortality worldwide for 36 cancers in 185 countries. CA Cancer J Clin (2021) 71(3):209–49. doi: 10.3322/caac.21660 33538338

[2] Li S Young KH Medeiros LJ. Diffuse large B-cell lymphoma. Pathology (2018) 50(1):74–87. doi: 10.1016/j.pathol.2017.09.006 29167021

[3] Liu Y Barta SK. Diffuse large B-cell lymphoma: 2019 update on diagnosis, risk stratification, and treatment. Am J Hematol (2019) 94(5):604–16. doi: 10.1002/ajh.25460 30859597

[4] Huang T Song X Xu D Tiek D Goenka A Wu B. Stem cell programs in cancer initiation, progression, and therapy resistance. Theranostics (2020) 10(19):8721–43. doi: 10.7150/thno.41648 PMC7392012 32754274

[5] Yang H Wang L Zheng Y Hu G Ma H Shen L. Knockdown of zinc finger protein 267 suppresses diffuse large B-cell lymphoma progression, metastasis, and cancer stem cell properties. Bioengineered (2022) 13(1):1686–701. doi: 10.1080/21655979.2021.2014644 PMC8805851 35001816

[6] Zhu P Fan Z. Cancer stem cells and tumorigenesis. Biophys Rep (2018) 4(4):178–88. doi: 10.1007/s41048-018-0062-2 PMC6153490 30310855

[7] Pan XW Zhang H Xu D Chen JX Chen WJ Gan SS. Identification of a novel cancer stem cell subpopulation that promotes progression of human fatal renal cell carcinoma by single-cell rna-seq analysis. Int J Biol Sci (2020) 16(16):3149–62. doi: 10.7150/ijbs.46645 PMC7645996 33162821

[8] Chen J Ge X Zhang W Ding P Du Y Wang Q. Pi3k/akt inhibition reverses R-chop resistance by destabilizing sox2 in diffuse large B cell lymphoma. Theranostics (2020) 10(7):3151–63. doi: 10.7150/thno.41362 PMC7053184 32194860

[9] Gross E Quillet-Mary A Ysebaert L Laurent G Fournie JJ. Cancer stem cells of differentiated B-cell Malignancies: models and consequences. Cancers (Basel) (2011) 3(2):1566–79. doi: 10.3390/cancers3021566 PMC3757378 24212774

[10] Martinez-Climent JA Fontan L Gascoyne RD Siebert R Prosper F. Lymphoma stem cells: enough evidence to support their existence? Haematologica (2010) 95(2):293–302. doi: 10.3324/haematol.2009.013318 20139392 PMC2817033

[11] Baslan T Hicks J. Unravelling biology and shifting paradigms in cancer with single-cell sequencing. Nat Rev Cancer (2017) 17(9):557–69. doi: 10.1038/nrc.2017.58 28835719

[12] Lei Y Tang R Xu J Wang W Zhang B Liu J. Applications of single-cell sequencing in cancer research: progress and perspectives. J Hematol Oncol (2021) 14(1):91. doi: 10.1186/s13045-021-01105-2 34108022 PMC8190846

[13] Steen CB Luca BA Esfahani MS Azizi A Sworder BJ Nabet BY. The landscape of tumor cell states and ecosystems in diffuse large B cell lymphoma. Cancer Cell (2021) 39(10):1422–37 e10. doi: 10.1016/j.ccell.2021.08.011 34597589 PMC9205168

[14] Hao Y Hao S Andersen-Nissen E Mauck WM 3rd Zheng S Butler A. Integrated analysis of multimodal single-cell data. Cell (2021) 184(13):3573–87 e29. doi: 10.1016/j.cell.2021.04.048 34062119 PMC8238499

[15] Korsunsky I Millard N Fan J Slowikowski K Zhang F Wei K. Fast, sensitive and accurate integration of single-cell data with harmony. Nat Methods (2019) 16(12):1289–96. doi: 10.1038/s41592-019-0619-0 PMC6884693 31740819

[16] Gao R Bai S Henderson YC Lin Y Schalck A Yan Y. Delineating copy number and clonal substructure in human tumors from single-cell transcriptomes. Nat Biotechnol (2021) 39(5):599–608. doi: 10.1038/s41587-020-00795-2 33462507 PMC8122019

[17] Cao J Spielmann M Qiu X Huang X Ibrahim DM Hill AJ. The single-cell transcriptional landscape of mammalian organogenesis. Nature (2019) 566(7745):496–502. doi: 10.1038/s41586-019-0969-x 30787437 PMC6434952

[18] Newman AM Steen CB Liu CL Gentles AJ Chaudhuri AA Scherer F. Determining cell type abundance and expression from bulk tissues with digital cytometry. Nat Biotechnol (2019) 37(7):773–82. doi: 10.1038/s41587-019-0114-2 PMC6610714 31061481

[19] Hanzelmann S Castelo R Guinney J. Gsva: gene set variation analysis for microarray and rna-seq data. BMC Bioinf (2013) 14:7. doi: 10.1186/1471-2105-14-7 PMC3618321 23323831

[20] Aibar S Gonzalez-Blas CB Moerman T Huynh-Thu VA Imrichova H Hulselmans G. Scenic: single-cell regulatory network inference and clustering. Nat Methods (2017) 14(11):1083–6. doi: 10.1038/nmeth.4463 PMC5937676 28991892

[21] Vento-Tormo R Efremova M Botting RA Turco MY Vento-Tormo M Meyer KB. Single-cell reconstruction of the early maternal-fetal interface in humans. Nature (2018) 563(7731):347–53. doi: 10.1038/s41586-018-0698-6 PMC7612850 30429548

[22] Wu T Hu E Xu S Chen M Guo P Dai Z. Clusterprofiler 4.0: A universal enrichment tool for interpreting omics data. Innovation (Camb) (2021) 2(3):100141. doi: 10.1016/j.xinn.2021.100141 34557778 PMC8454663

[23] Ye X Wang L Nie M Wang Y Dong S Ren W. A single-cell atlas of diffuse large B cell lymphoma. Cell Rep (2022) 39(3):110713. doi: 10.1016/j.celrep.2022.110713 35443163

[24] Van de Sande B Flerin C Davie K De Waegeneer M Hulselmans G Aibar S. A scalable scenic workflow for single-cell gene regulatory network analysis. Nat Protoc (2020) 15(7):2247–76. doi: 10.1038/s41596-020-0336-2 32561888

[25] Winkelmann R Sandrock L Kirberg J Jack HM Schuh W. Klf2–a negative regulator of pre-B cell clonal expansion and B cell activation. PloS One (2014) 9(5):e97953. doi: 10.1371/journal.pone.0097953 24874925 PMC4038547

[26] Luo Y Xu WB Ma B Wang Y. Novel stemness-related gene signature predicting prognosis and indicating a different immune microenvironment in hnscc. Front Genet (2022) 13:822115. doi: 10.3389/fgene.2022.822115 35360859 PMC8963956

[27] Dhanasekaran R Deutzmann A Mahauad-Fernandez WD Hansen AS Gouw AM Felsher DW. The myc oncogene - the grand orchestrator of cancer growth and immune evasion. Nat Rev Clin Oncol (2022) 19(1):23–36. doi: 10.1038/s41571-021-00549-2 34508258 PMC9083341

[28] Karadkhelkar NM Lin M Eubanks LM Janda KD. Demystifying the druggability of the myc family of oncogenes. J Am Chem Soc (2023) 145(6):3259–69. doi: 10.1021/jacs.2c12732 PMC10182829 36734615

[29] Savage KJ Johnson NA Ben-Neriah S Connors JM Sehn LH Farinha P. Myc gene rearrangements are associated with a poor prognosis in diffuse large B-cell lymphoma patients treated with R-chop chemotherapy. Blood (2009) 114(17):3533–7. doi: 10.1182/blood-2009-05-220095 19704118

[30] Barrans S Crouch S Smith A Turner K Owen R Patmore R. Rearrangement of myc is associated with poor prognosis in patients with diffuse large B-cell lymphoma treated in the era of rituximab. J Clin Oncol (2010) 28(20):3360–5. doi: 10.1200/JCO.2009.26.3947 20498406

[31] Copie-Bergman C Cuilliere-Dartigues P Baia M Briere J Delarue R Canioni D. Myc-ig rearrangements are negative predictors of survival in dlbcl patients treated with immunochemotherapy: A gela/lysa study. Blood (2015) 126(22):2466–74. doi: 10.1182/blood-2015-05-647602 26373676

[32] Kumar A Taghi Khani A Duault C Aramburo S Sanchez Ortiz A Lee SJ. Intrinsic suppression of type I interferon production underlies the therapeutic efficacy of il-15-producing natural killer cells in B-cell acute lymphoblastic leukemia. J Immunother Cancer (2023) 11(5):e006649. doi: 10.1136/jitc-2022-006649 37217248 PMC10231005

[33] Shah K Al-Haidari A Sun J Kazi JU. T cell receptor (Tcr) signaling in health and disease. Signal Transduct Target Ther (2021) 6(1):412. doi: 10.1038/s41392-021-00823-w 34897277 PMC8666445

[34] Ruan X Yi J Hu L Zhi J Zeng Y Hou X. Reduced mhc class ii expression in medullary thyroid cancer identifies patients with poor prognosis. Endocr Relat Cancer (2022) 29(2):87–98. doi: 10.1530/ERC-21-0153 34874277

[35] Liu W Hu H Shao Z Lv X Zhang Z Deng X. Characterizing the tumor microenvironment at the single-cell level reveals a novel immune evasion mechanism in osteosarcoma. Bone Res (2023) 11(1):4. doi: 10.1038/s41413-022-00237-6 36596773 PMC9810605

[36] Fang Y Wang L Wan C Sun Y van der Jeught K Zhou Z. Mal2 drives immune evasion in breast cancer by suppressing tumor antigen presentation. J Clin Invest (2021) 131(1):e140837. doi: 10.1172/JCI140837 32990678 PMC7773365

[37] Yamamoto K Venida A Yano J Biancur DE Kakiuchi M Gupta S. Autophagy promotes immune evasion of pancreatic cancer by degrading mhc-I. Nature (2020) 581(7806):100–5. doi: 10.1038/s41586-020-2229-5 PMC7296553 32376951

[38] Zhang B Li J Hua Q Wang H Xu G Chen J. Tumor cemip drives immune evasion of colorectal cancer *via* mhc-I internalization and degradation. J Immunother Cancer (2023) 11(1):e005592. doi: 10.1136/jitc-2022-005592 36596591 PMC9815088

[39] Zhan L Zhang J Zhang J Liu X Zhu S Shi Y. Lc3 and nlrc5 interaction inhibits nlrc5-mediated mhc class I antigen presentation pathway in endometrial cancer. Cancer Lett (2022) 529:37–52. doi: 10.1016/j.canlet.2021.12.031 34974132

[40] Angell TE Lechner MG Jang JK LoPresti JS Epstein AL. Mhc class I loss is a frequent mechanism of immune escape in papillary thyroid cancer that is reversed by interferon and selumetinib treatment in vitro. Clin Cancer Res (2014) 20(23):6034–44. doi: 10.1158/1078-0432.CCR-14-0879 PMC4252612 25294906

[41] Gettinger S Choi J Hastings K Truini A Datar I Sowell R. Impaired hla class I antigen processing and presentation as a mechanism of acquired resistance to immune checkpoint inhibitors in lung cancer. Cancer Discovery (2017) 7(12):1420–35. doi: 10.1158/2159-8290.CD-17-0593 PMC5718941 29025772

[42] Fangazio M Ladewig E Gomez K Garcia-Ibanez L Kumar R Teruya-Feldstein J. Genetic mechanisms of hla-I loss and immune escape in diffuse large B cell lymphoma. Proc Natl Acad Sci U.S.A. (2021) 118(22):e2104504118. doi: 10.1073/pnas.2104504118 34050029 PMC8179151

[43] Patel N Slack GW Bodo J Ben-Neriah S Villa D Durkin L. Immune escape mechanisms in intravascular large B-cell lymphoma: A molecular cytogenetic and immunohistochemical study. Am J Clin Pathol (2022) 157(4):578–85. doi: 10.1093/ajcp/aqab154 34724028

[44] Lambert SA Jolma A Campitelli LF Das PK Yin Y Albu M. The human transcription factors. Cell (2018) 172(4):650–65. doi: 10.1016/j.cell.2018.01.029 PMC12908702 29425488

[45] Yuan L Tian X Zhang Y Huang X Li Q Li W. Linc00319 promotes cancer stem cell-like properties in laryngeal squamous cell carcinoma via E2f1-mediated upregulation of hmgb3. Exp Mol Med (2021) 53(8):1218–28. doi: 10.1038/s12276-021-00647-2 PMC8417254 34408262

[46] Gao S Zhang X Bai W Wang J Jiang B. Circ-igf1r affects the progression of colorectal cancer by activating the mir-362-5p/hmgb3-mediated wnt/beta-catenin signal pathway. Biochem Genet (2023) 61(3):1210–29. doi: 10.1007/s10528-022-10316-2 36542208

[47] Xiao B Lv SG Wu MJ Shen XL Tu W Ye MH. Circ_Clip2 promotes glioma progression through targeting the mir-195-5p/hmgb3 axis. J Neurooncol (2021) 154(2):131–44. doi: 10.1007/s11060-021-03814-7 34357490

[48] Sichtig N Korfer N Steger G. Papillomavirus binding factor binds to sap30 and represses transcription via recruitment of the hdac1 co-repressor complex. Arch Biochem Biophys (2007) 467(1):67–75. doi: 10.1016/j.abb.2007.08.015 17897615

[49] Chen Z Zou Y Zhang Y Chen Z Wu F Shi N. A novel prognostic signature based on four glycolysis-related genes predicts survival and clinical risk of hepatocellular carcinoma. J Clin Lab Anal (2021) 35(11):e24005. doi: 10.1002/jcla.24005 34523732 PMC8605142

[50] Hu CL Chen BY Li Z Yang T Xu CH Yang R. Targeting uhrf1-sap30-mxd4 axis for leukemia initiating cell eradication in myeloid leukemia. Cell Res (2022) 32(12):1105–23. doi: 10.1038/s41422-022-00735-6 PMC9715639 36302855

[51] Ren B Cam H Takahashi Y Volkert T Terragni J Young RA. E2f integrates cell cycle progression with DNA repair, replication, and G(2)/M checkpoints. Genes Dev (2002) 16(2):245–56. doi: 10.1101/gad.949802 PMC155321 11799067

[52] DeGregori J Johnson DG. Distinct and overlapping roles for E2f family members in transcription, proliferation and apoptosis. Curr Mol Med (2006) 6(7):739–48. doi: 10.2174/1566524010606070739 17100600

[53] Weijts BG Bakker WJ Cornelissen PW Liang KH Schaftenaar FH Westendorp B. E2f7 and E2f8 promote angiogenesis through transcriptional activation of vegfa in cooperation with hif1. EMBO J (2012) 31(19):3871–84. doi: 10.1038/emboj.2012.231 PMC3463843 22903062

[54] Zhang Z Li J Huang Y Peng W Qian W Gu J. Upregulated mir-1258 regulates cell cycle and inhibits cell proliferation by directly targeting E2f8 in crc. Cell Prolif (2018) 51(6):e12505. doi: 10.1111/cpr.12505 30144184 PMC6528920

[55] Sun J Shi R Zhao S Li X Lu S Bu H. E2f8, a direct target of mir-144, promotes papillary thyroid cancer progression via regulating cell cycle. J Exp Clin Cancer Res (2017) 36(1):40. doi: 10.1186/s13046-017-0504-6 28270228 PMC5341194

[56] Yang AP Liu LG Chen MM Liu F You H Liu L. Integrated analysis of 10 lymphoma datasets identifies E2f8 as a key regulator in burkitt’s lymphoma and mantle cell lymphoma. Am J Transl Res (2019) 11(7):4382–96. PMC6684893 31396343

[57] Taghehchian N Maharati A Akhlaghipour I Zangouei AS Moghbeli M. Prc2 mediated klf2 down regulation: A therapeutic and diagnostic axis during tumor progression. Cancer Cell Int (2023) 23(1):233. doi: 10.1186/s12935-023-03086-3 37807067 PMC10561470

[58] Dostert C Grusdat M Letellier E Brenner D. The tnf family of ligands and receptors: communication modules in the immune system and beyond. Physiol Rev (2019) 99(1):115–60. doi: 10.1152/physrev.00045.2017 30354964

[59] Hahne M Kataoka T Schroter M Hofmann K Irmler M Bodmer JL. April, a new ligand of the tumor necrosis factor family, stimulates tumor cell growth. J Exp Med (1998) 188(6):1185–90. doi: 10.1084/jem.188.6.1185 PMC2212534 9743536

[60] Ullah MA Mackay F. The baff-april system in cancer. Cancers (Basel) (2023) 15(6):1791. doi: 10.3390/cancers15061791 36980677 PMC10046288

[61] Steinberg MW Cheung TC Ware CF. The signaling networks of the herpesvirus entry mediator (Tnfrsf14) in immune regulation. Immunol Rev (2011) 244(1):169–87. doi: 10.1111/j.1600-065X.2011.01064.x PMC3381650 22017438

