# Supplementary material for: Unraveling the enigma of B cells in diffuse large B-cell lymphoma: unveiling cancer stem cell-like B cell subpopulation at single-cell resolution
Source: Front Immunol. 2023 Dec 11;14:1310292. doi: 10.3389/fimmu.2023.1310292 (PMC10750418; doi:10.3389/fimmu.2023.1310292)
Supplement: Supplementary file 1 [file DataSheet_1.pdf]

Supplementary Material

1 Supplementary Figures

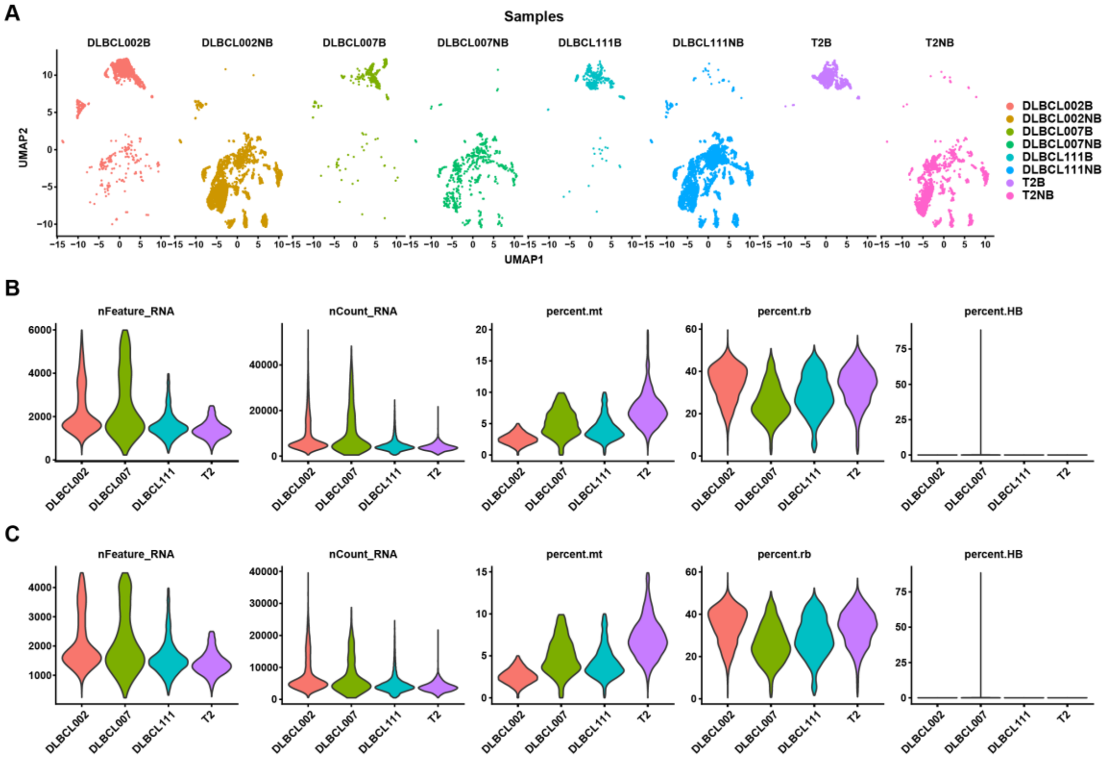

Supplementary Figure 1: Quantity control for single-cell RNA sequencing data. **(A)** UMAP embedding of cells from scRNA-seq data. A total of eight individual datasets were included, derived from four samples. Specifically, DLBCL002B and DLBCL002NB originated from the DLBCL002 sample, DLBCL007B and DLBCL007NB originated from the DLBCL007 sample, DLBCL111B and DLBCL111NB originated from the DLBCL111 sample, and T2B and T2NB originated from the T2 sample. These datasets were obtained through flow cytometry-based cell sorting, which enabled the selection of datasets containing B cell subsets (B) and datasets devoid of B cell subsets (NB). **(B)** Violin plot sequentially showing the number of transcripts, unique molecular identifiers (UMIs), the percent of mitochondrial genes, ribosome genes, and RBC genes per cell before quantity control. **(C)** Violin plot sequentially showing the number of transcripts, unique molecular identifiers (UMIs), the percent of mitochondrial genes, ribosome genes, and RBC genes per cell after quantity control.

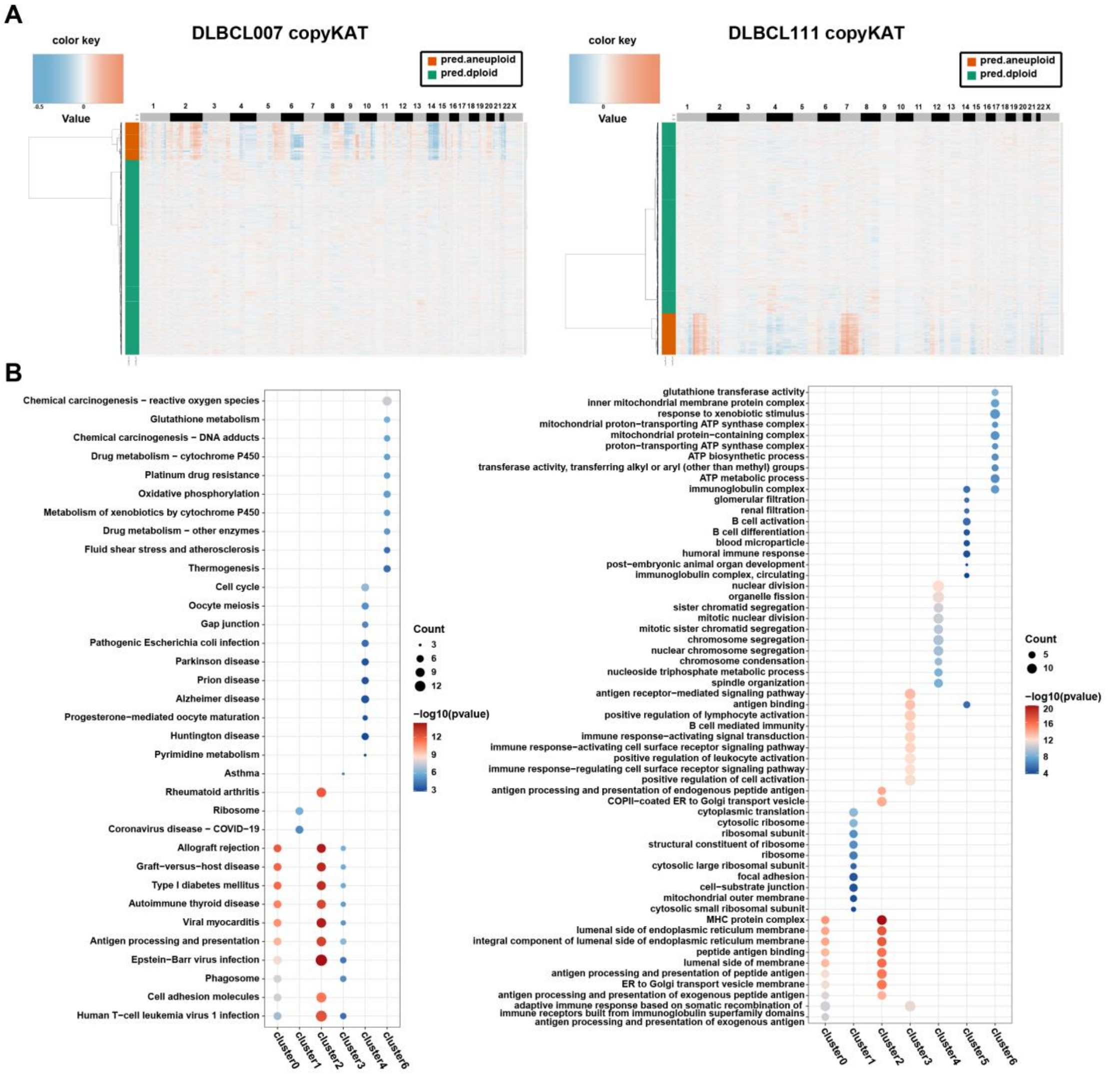

Supplementary Figure 2: CopyKAT and functional enrichment analysis in B cell subsets. (A) The chromatin deletion and amplification in benign (green) and malignant (orange) B cell subsets in the DLBCL007 and DLBCL111 samples. **(B)** Gene enrichment analysis was performed on the differentially expressed genes of the seven B cell subsets using the KEGG gene sets (left panel) and GO gene sets (right panel).

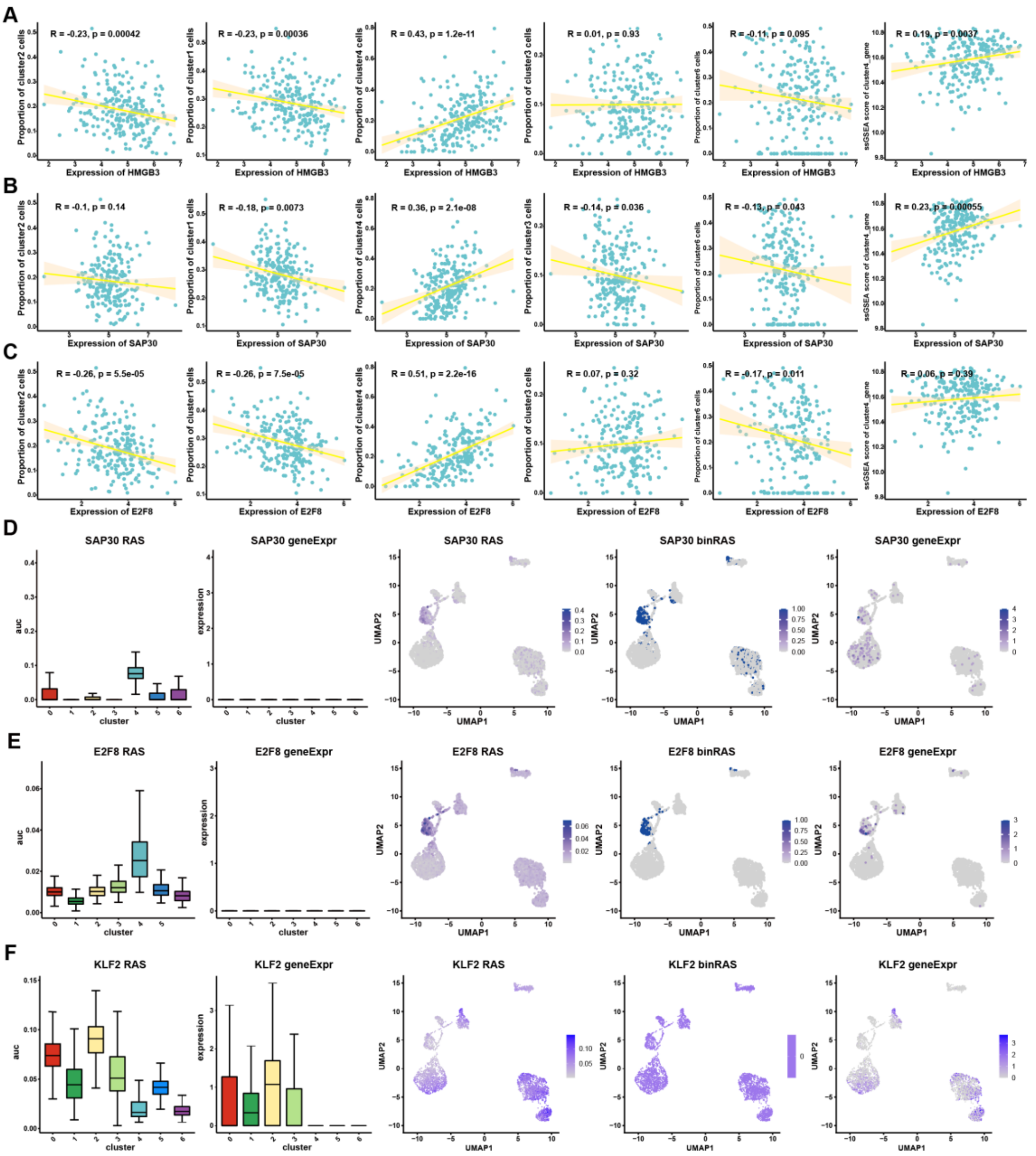

Supplementary Figure 3: Transcription factor regulatory networks and correlation between B cell subsets infiltration with the expression of transcription factors. **(A)** Correlation between B cell subsets infiltration abundance with the expression of *HMGB3*. **(B)** Correlation between B cell subsets infiltration abundance with the expression of *SAP30*. **(C)** Correlation between B cell subsets infiltration abundance with the expression of *E2F8*. **(D)** Expression and RAS (regulon activity score) of *SAP30* in seven B cell subsets. **(E)** Expression and RAS of *E2F8* in seven B cell subsets. **(F)** Expression and RAS of *KLF2* in seven B cell subsets.

2 Supplementary Tables

Table S1: Differential expression genes of B cell subgroups.
Table S2: The activity of transcription factor regulatory networks in B cell subgroups.
Table S3: The regulatory specificity score (RSS) of B cell subgroups.
Table S4: Specific genes of cluster 4 for ssGSEA.
